# Expression of toll like receptor 8 (TLR8) in specific groups of mouse hippocampal interneurons

**DOI:** 10.1371/journal.pone.0267860

**Published:** 2022-05-04

**Authors:** Lennart Seizer, Sadegh Rahimi, Sandra Santos-Sierra, Meinrad Drexel

**Affiliations:** 1 Department of Pharmacology, Medical University of Innsbruck, Innsbruck, Austria; 2 Institute of Psychology, University of Innsbruck, Innsbruck, Austria; University of Modena and Reggio Emilia, ITALY

## Abstract

Toll-like receptors (TLR) are one of the main constituents of the innate immune system in mammals. They can detect conserved microbial structures (pathogen-associated molecular patterns) and host-derived ligands that are produced during cellular stress and damage (danger-associated molecular patterns) and may then initiate an intracellular signaling cascade leading to the expression of pro-inflammatory cytokines and immediate immune responses. Some TLR (TLR1, 2, 4, 5, and 6) are expressed on the cell surface while others (TLR3, 7, 8 and 9) are present on the surface of endosomes and their ligands require internalization before recognition is possible. Several TLR have also been detected in neurons where they may serve functions that are not related to immune responses. TLR2, 3, and 4 have been described in cortical neurons and, for TLR4, a seizure-promoting role in epilepsies associated with inflammation has been shown. TLR3, 7, and 8 expressed in neurons seem to influence the growth or withdrawal of neurites and robust activation of TLR8 in neurons may even induce neuronal death. The goal of the current study was to investigate the expression of TLR8 in the hippocampus of mice during postnatal development and in adulthood. We focused on three functionally distinct groups of GABAergic interneurons characterized by the expression of the molecular markers parvalbumin, somatostatin, or calretinin, and we applied double fluorescence immunohistochemistry and cell counts to quantify co-expression of TLR8 in the three groups of GABA-interneurons across hippocampal subregions. We found subregion-specific differences in the expression of TLR8 in these interneurons. During postnatal development, TLR8 was detected only in mice older than P5. While only a small fraction of hippocampal calretinin-positive interneurons expressed TLR8, most parvalbumin-positive interneurons in all hippocampal subregions co-expressed TLR8. Somatostatin-positive interneurons co-expressing TLR8 were mainly present in hippocampal sector CA3 but rare in the dentate gyrus and CA1. High expression of TLR8 in parvalbumin-interneurons may contribute to their high vulnerability in human temporal lobe epilepsy.

## Introduction

Toll-like receptors (TLR) are mammalian orthologues of a transmembrane receptor first discovered in *Drosophila melanogaster*, which takes part in establishing body axis polarity during embryonic development and is necessary to launch an innate immune response upon fungal infection in an adult fly [1,2]. In mammals, TLRs are main constituents of the innate immune system and play a major role in generating immune responses because of their ability to recognize conserved microbial structures, so-called pathogen-associated molecular patterns (PAMPs). They also recognize host-derived ligands produced during cellular stress and damage (danger-associated molecular patterns: DAMPs) like high mobility group-1 (HMGB-1), single-stranded RNA (ssRNA) or hyaluron. Ligand recognition activates intracellular signaling pathways, which induce the expression of several pro-inflammatory cytokines, thereby launching immediate innate responses. In turn, antigen-presenting cells (e.g., macrophages and dendritic cells) become activated what initiates adaptive immune responses [3]. TLRs may be expressed at the plasma membrane or in the endosomal compartments on various immune cells and even on non-immune cells, such as fibroblasts and epithelial cells. While TLR1, 2, 4, 5, and 6 are expressed on the cell surface, TLR3, 7, 8, and 9 are almost exclusively found in endosomes and their ligands require internalization before recognition and subsequent signaling is possible.

The TLR7 and TLR8 genes are located on the X chromosome and show high homology. Mouse TLR7 and human TLR8 recognize ssRNA of viral and host origin, synthetic imidazoquinoline molecules (e.g., resiquimod) and some guanine nucleotide analogs. Both, TLR7 and TLR8 are expressed in mice, but mouse TLR8 seemed to be nonfunctional [4,5].

Besides their well-studied immunological role, several TLRs with poorly characterized function have been found in neurons as well. For example, TLR2 and 4 are expressed in neuronal progenitor cells (NPCs) [6] and, together with TLR3, in cortical neurons [7]. While TLR2 was also detected in post-ischemic brain tissue [8], TLR3 was found in human neurons [9] and TLR4 in trigeminal sensory neurons [10] and dorsal root ganglion neurons [11].

Additionally to PAMPs, ligands of viral and bacterial origin, endosomal TLRs, like TLR3, TLR7 and TLR8, can recognize host-derived mRNA and DNA, which could act as intrinsic signals [12,13]. This way, TLR systems would enable neurons to detect exogenous as well as intrinsic signals to regulate their growth and differentiation [14]. For instance, TLR3 and TLR7 have been shown to cause dendrites and axons to withdraw via *c-Fos* expression and IL-6 production or by downregulating *Disc1* [15–17].

This study focuses on TLR8, probably the least studied among the different TLRs. Ma et al. (2006) found TLR8 to act as a negative regulator of neurite growth in neurons. Activation of TLR8 with resiquimod (R-848), a TLR7/8 ligand, results in significantly reduced primary neurites and can dose-dependently induce neuronal death. On the other hand, inhibition of TLR8 leads to neurite elongation and reduced neuronal death. The expression of TLR8 changes during the period of neuronal differentiation and axonogenesis in mice. It is first abundant at embryonic day 12 and increases steadily until postnatal day 21, when it decreases again. During embryogenesis, TLR8 is present in axons, while postnatally it transfers to a diffuse expression in the soma, suggesting that TLR8 plays a role in neuronal development. Ma et al. [17,18] provided evidence of TLR8 functioning in neurons, without using the canonical (immunological) TLR signaling pathway, including NF-kB. TLR8 activation might operate through IkBa-mediated activation/repression of certain (yet unknown) genes, but the exact link leading to neuronal responses remains unclear.

Using double-immunohistochemistry for TLR8 and markers for glial cells and neuronal subgroups, we investigated the distribution of TLR8 in the mouse hippocampus at different developmental stages. We find differential expression of TLR8 in hippocampal neuronal populations in an age-related manner what may aid to understand the role of this bona fide innate immune receptor in neurons.

## Materials and methods

### Mice

**Fig 1:**
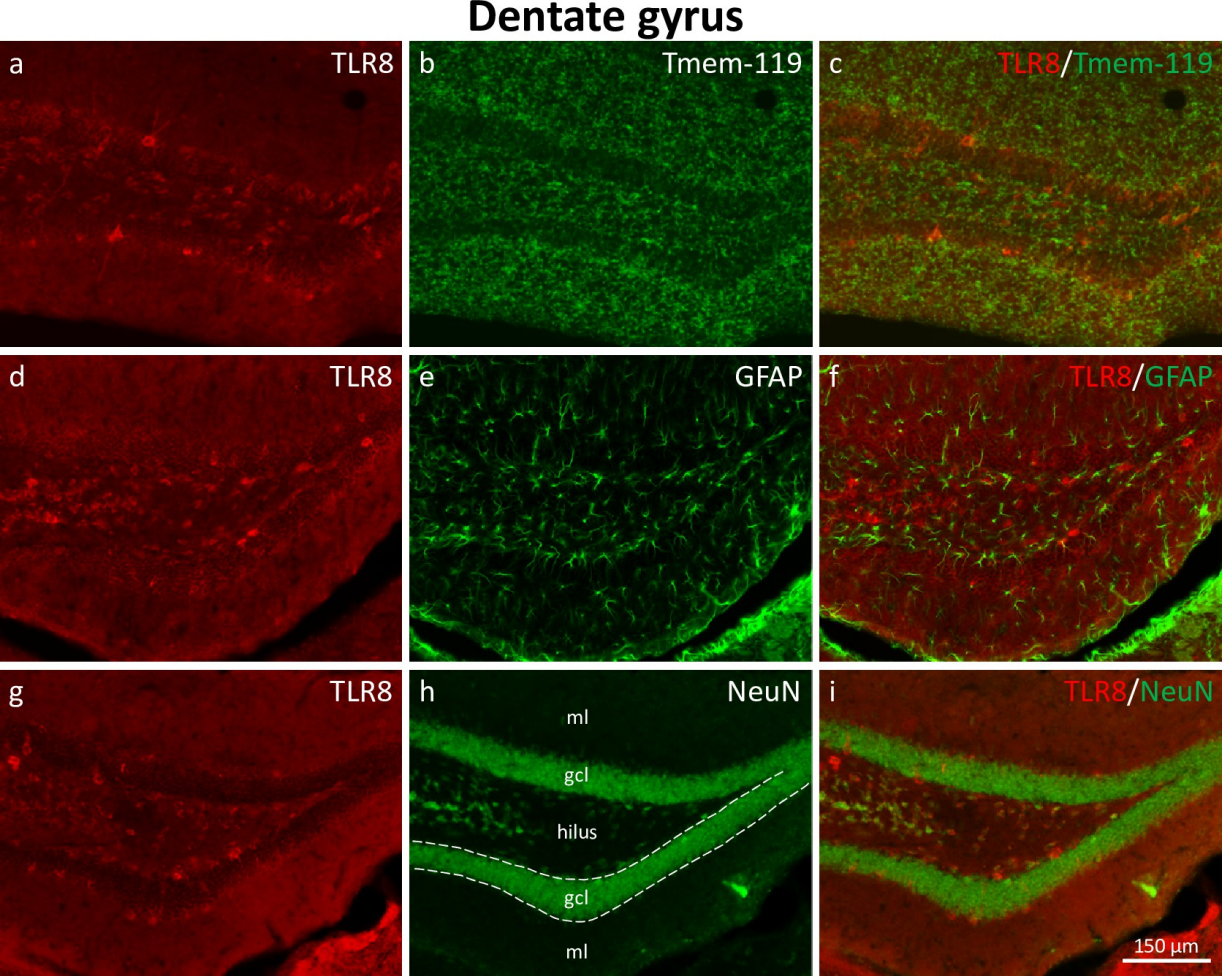
Expression of TLR8 in the dentate gyrus. Double immunofluorescence of TLR8 (red fluorescence; a, d, g) with the microglia marker transmembrane protein 119 (Tmem-119; b, c), the astrocyte marker glial fibrillary acidic protein (GFAP; e, f), and neuron-specific nuclear protein (NeuN; h, i), a marker for adult neurons. TLR8-immunoreactive cells were detected in the hilus of the dentate gyrus, in the supragranular layer, in the granule cell layer, and at the border between the granule cell layer and the inner molecular layer of the dentate gyrus (a, d, g). Cell bodies of granule cells showed only faint labeling. Double immunofluorescence revealed expression of TLR8 in neurons (NeuN, i), but not in glial cells (Tmem-119, c; GFAP, i). Abbreviations: gcl, granule cell layer; ml, molecular layer.

**Fig 2:**
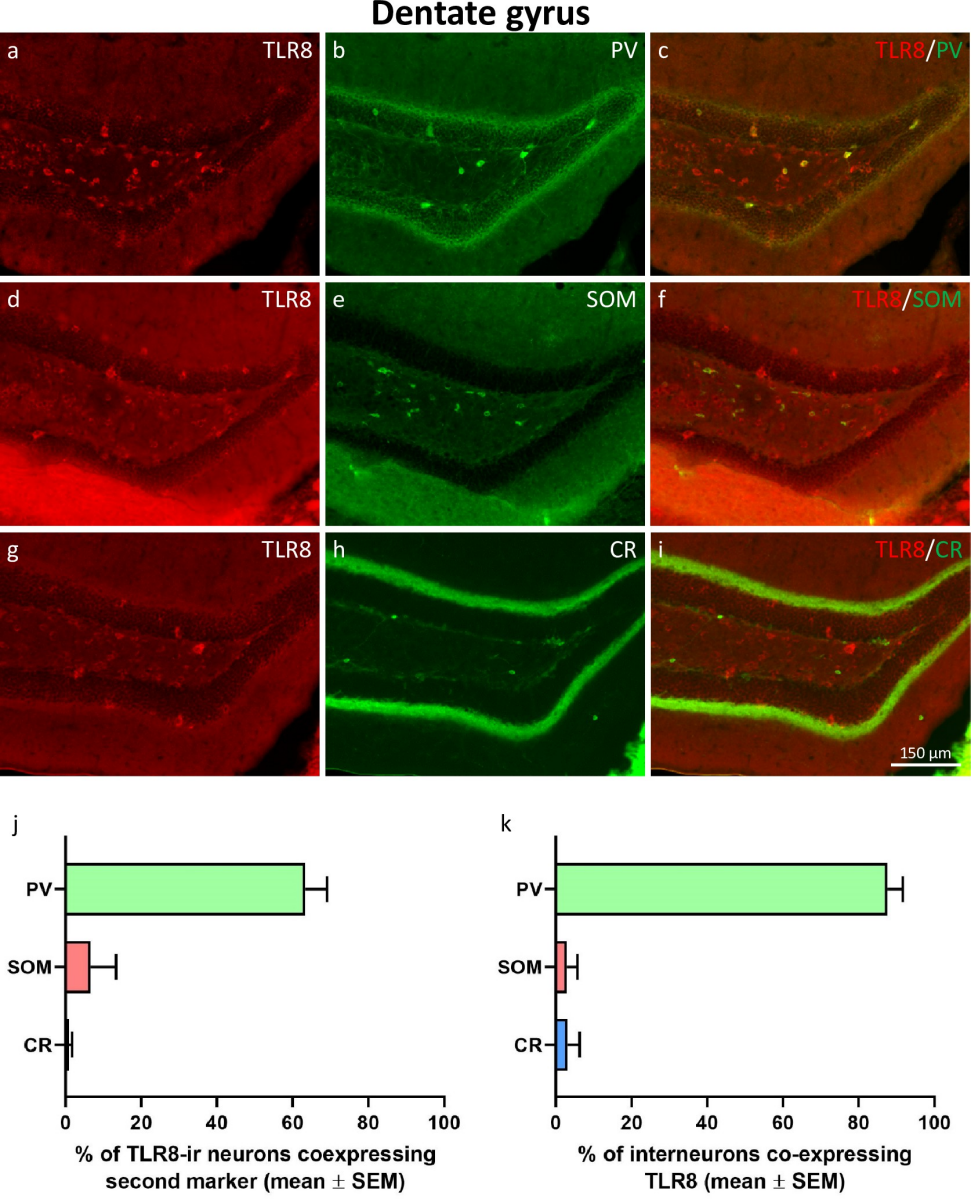
Expression of TLR8 in GABAergic interneurons of the dentate gyrus. Double immunofluorescence of TLR8 (red fluorescence; a, d, g) with parvalbumin (PV; b, c), somatostatin (SOM; e, f), and calretinin (CR; h, i). TLR8 was predominately expressed in PV-positive interneurons and to a minor extent in SOM- or CR-expressing GABAergic interneurons. Quantification of double-labeling revealed that 63.4 ± 5.70% of TLR8-expressing neurons also expressed PV, but only 6.7 ± 6.67% expressed SOM, and 0.9 ± 0.86% expressed CR (j). Vice versa, 87.7 ± 4.04% of PV-interneurons co-expressed TLR8, but only 2.9 ± 2.86% and 3.1 ± 3.13% of SOM- and CR-interneurons co-expressed TLR8, respectively.

**Fig 3:**
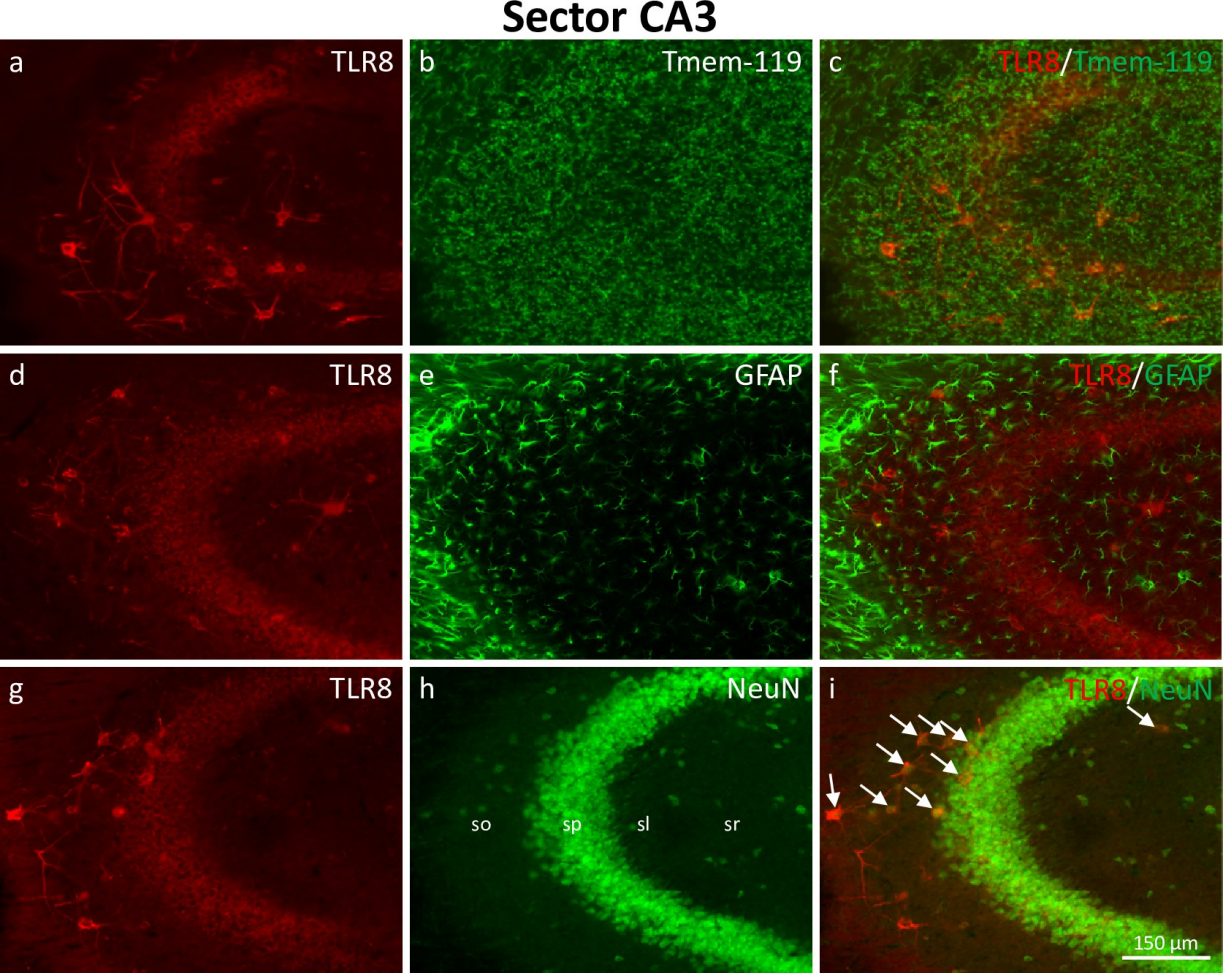
Expression of TLR8 in hippocampal sector CA3. Double-immunofluorescence showing TLR8 together with the glial markers Tmem-119 (microglia, a-c) and GFAP (astrocytes, d-f) and with the neuronal marker NeuN (g-i). In sector CA3 of the hippocampus proper, GABAergic interneurons strongly expressing TLR8 were located in stratum oriens, stratum pyramidale, stratum lucidum, and stratum radiatum (a, d, g). Weak expression of TLR8 was also present in glutamatergic pyramidal cells in stratum pyramidale. Also, in sector CA3, expression of TLR8 was restricted to neurons (see arrows in i); microglia and astrocytes did not express TLR8. Abbreviations: so, stratum oriens; sp, stratum pyramidale; sl, stratum lucidum; sr, stratum radiatum.

**Fig 4:**
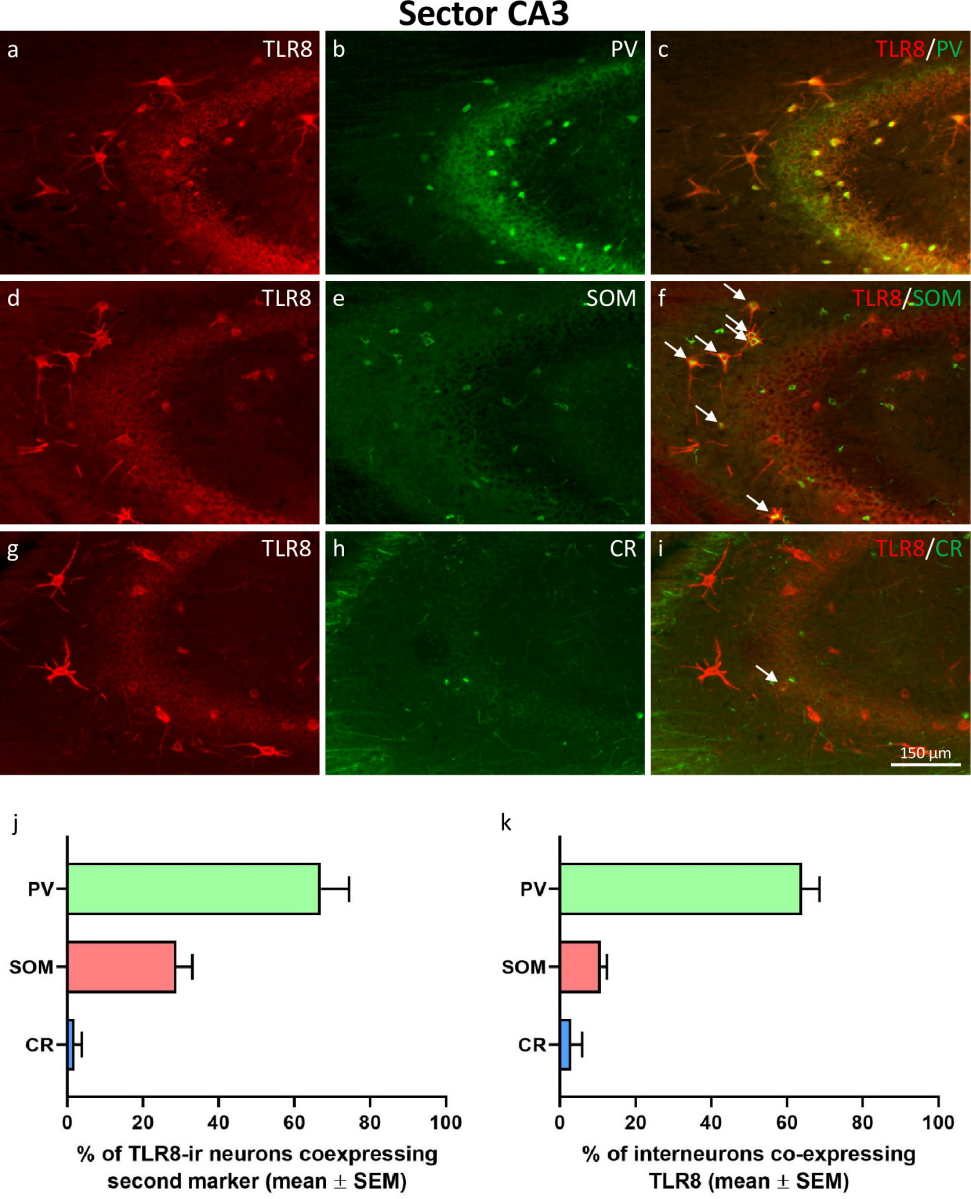
Expression of TLR8 in GABAergic interneurons of hippocampal sector CA3. Double-immunofluorescence of TLR8 together with parvalbumin (PV, a-c), somatostatin (SOM, d-f), and calretinin (CR, g-i). TLR8 was co-expressed in PV-ir interneurons in stratum oriens, stratum pyramidale, stratum lucidum, and stratum radiatum (c). Co-expression of TLR8 together with SOM seemed to be restricted to SOM-interneurons located in stratum oriens (see arrows in f) while SOM-interneurons located in stratum pyramidale, stratum lucidum, and stratum radiatum did not co-express TLR8. Only a fraction of CR-expressing interneurons in sector CA3 co-expressed TLR8 (g–I; see arrow in i). Quantification of co-labeling revealed that 67.0 ± 7.49% of TLR8-interneurons also expressed PV, 28.9 ± 4.19% co-expressed SOM, and only 1.9 ± 1.92% co-expressed CR (see panel j). Vice versa, quantification of co-labeling showed that 64.0 ± 4.56% of PV-interneurons, 10.7 ± 1.72% of SOM-interneurons, and only 2.9 ± 2.94% of CR-interneurons co-expressed TLR8 (see panel k).

**Fig 5:**
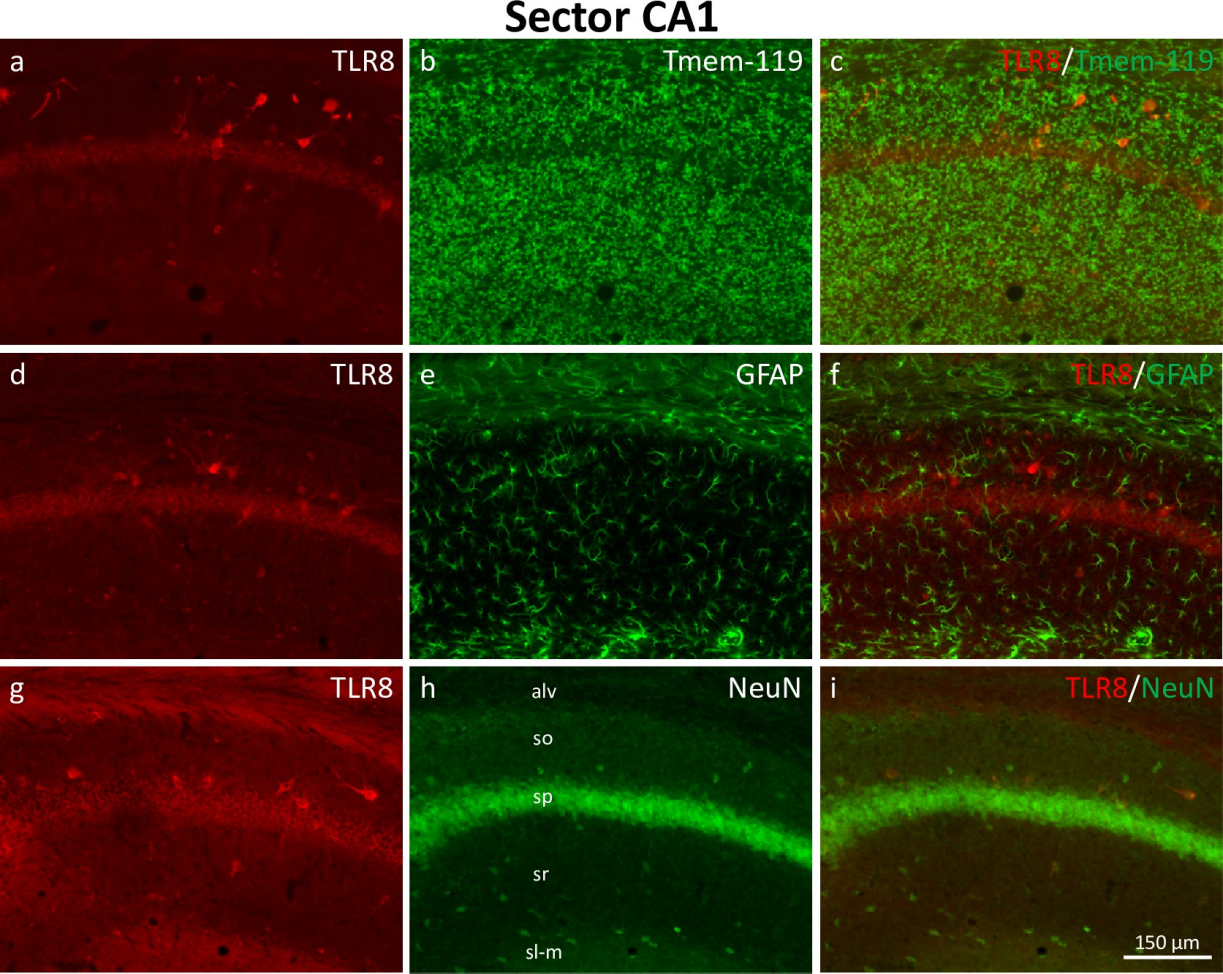
Expression of TLR8 in hippocampal sector CA1. Double-immunofluorescence of TLR8 together with Tmem-119 (microglial marker, a-c), GFAP (astrocyte marker, d-f), and NeuN (neuronal marker, g-i). TLR8 was expressed in presumed GABAergic interneurons in stratum oriens, stratum pyramidale, stratum radiatum, and stratum lacunosum-moleculare of sector CA1 (a, d, g). Weaker TLR8-labeling was present in CA1 principal neurons in stratum pyramidale. Co-labeling with Tmem-119, GFAP, and NeuN revealed that TLR8 expression in sector CA1 was restricted to neurons (i). Abbreviations: alv, alveus; so, stratum oriens; sp, stratum pyramidale; sr, stratum radiatum; sl-m, stratum lacunosum-moleculare.

**Fig 6:**
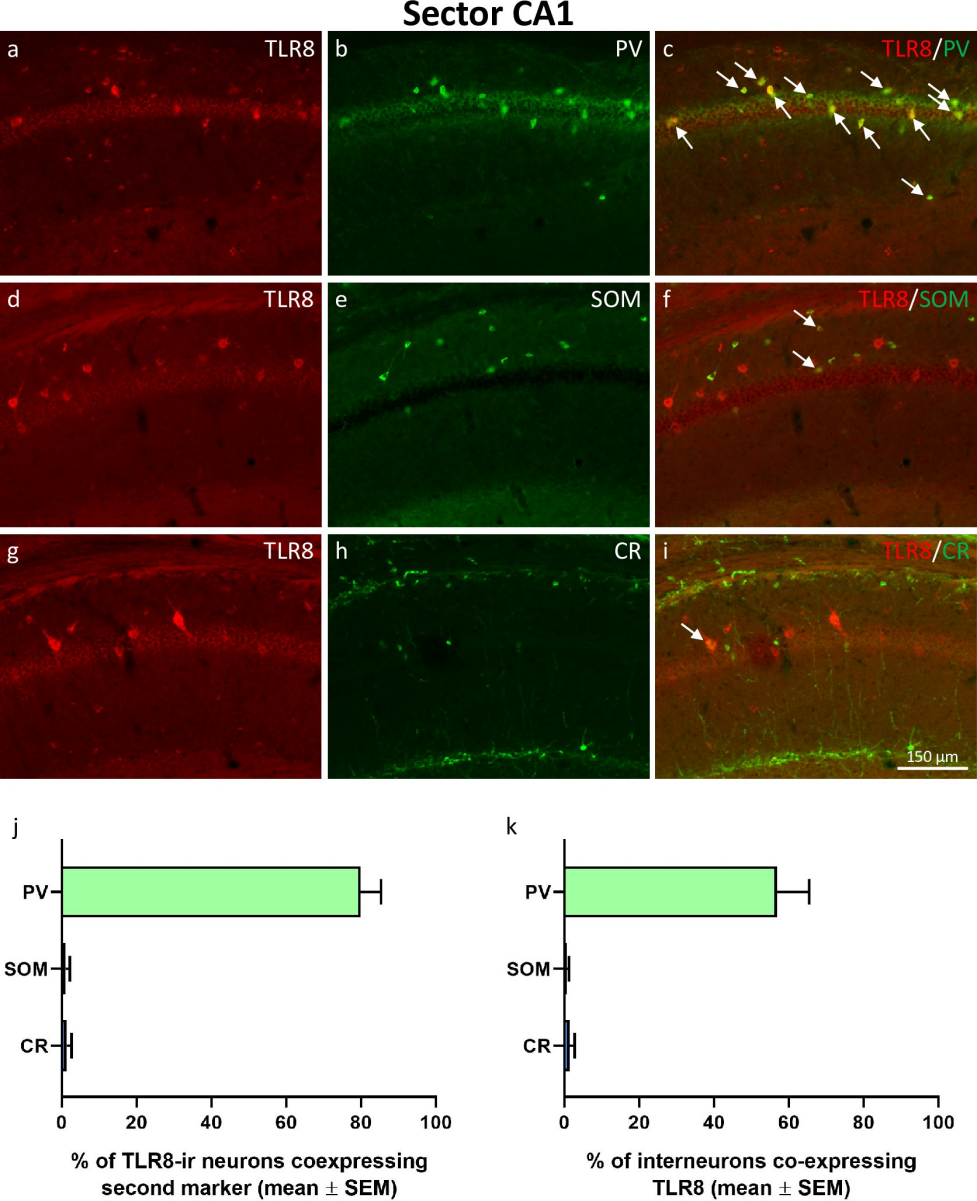
Expression of TLR8 in GABAergic interneurons of hippocampal sector CA1. Double-immunofluorescence of TLR8 together with parvalbumin (PV, a-c), somatostatin (SOM, d-f), and calretinin (CR, g-i). TLR8 was co-expressed in PV-ir interneurons in stratum oriens, stratum pyramidale, and at the border between stratum radiatum and stratum lacunosum-moleculare (c). Co-expression of TLR8 together with SOM was mainly present in a low number of SOM-interneurons in stratum oriens (see arrows in f). Co-labeling of TLR8 together with CR was rarely observed in sector CA1 (g-i; see arrow in i). Quantification of co-labeling revealed that 79.9 ± 5.47% of TLR8-interneurons also expressed PV, but only 1.1 ± 1.11% co-expressed SOM, and only 1.3 ± 1.32% co-expressed CR (see panel j). Vice versa, quantification of co-labeling showed that 56.8 ± 8.67% of PV-interneurons, 0.6 ± 0.61% of SOM-interneurons, and only 1.4 ± 1.39% of CR-interneurons co-expressed TLR8 (see panel k).

**Fig 7:**
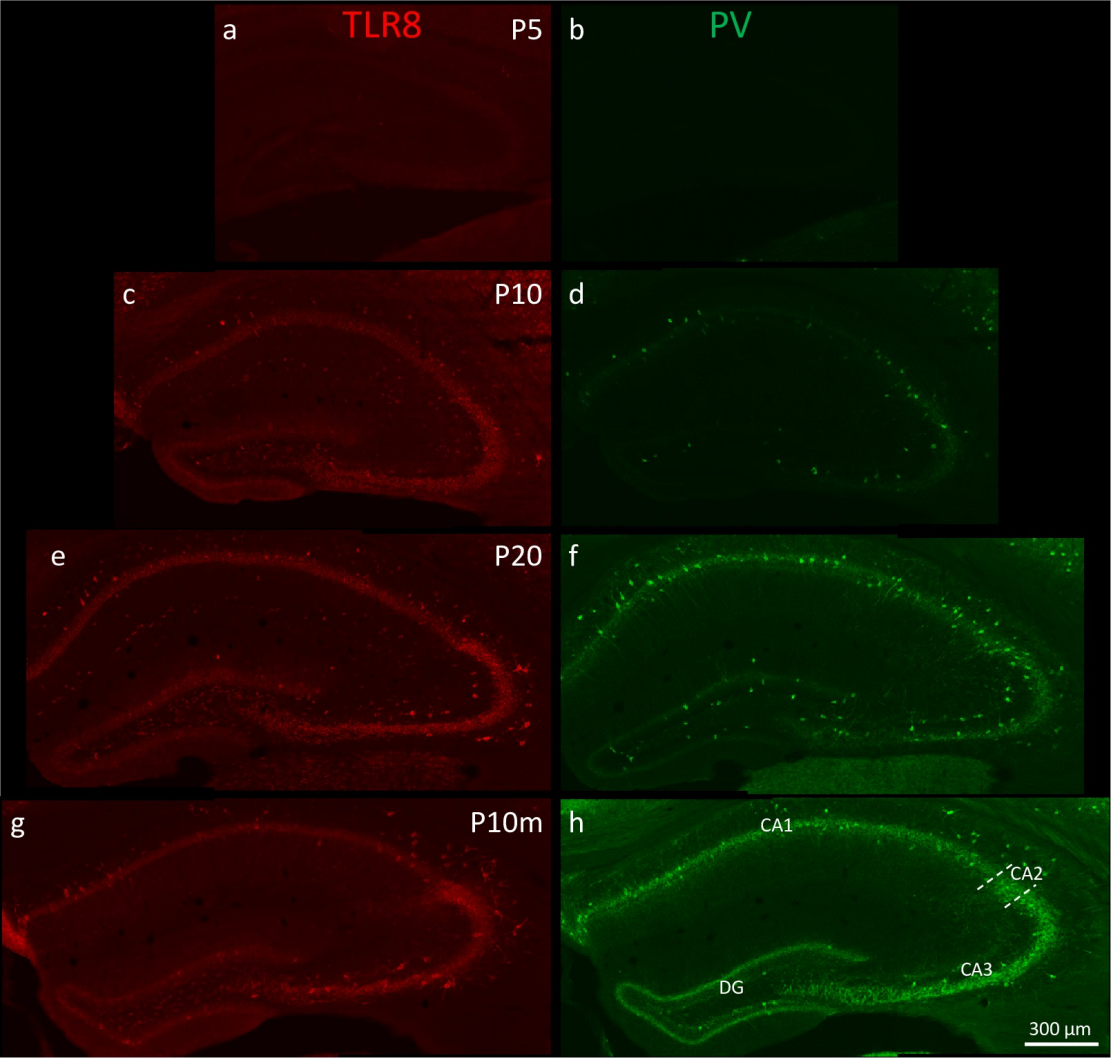
**Time-course of postnatal expression of TLR8 and PV in the mouse hippocampus.** At birth (P0), no TLR8- or PV-immunoreactivity was detected in the hippocampus (not shown). At P5, few TLR8-expressing interneurons, but no PV-expressing interneurons were visible (a, b). Numerous interneurons expressing TLR8 and/or PV were present in all subregions of the hippocampus at P10 (c, d). TLR8 was also expressed in granule cells of the dentate gyrus and in pyramidal neurons of the hippocampus proper. PV-labeling was restricted to cell bodies and neurites (dendrites; axon terminals surrounding somata of dentate granule cells and hippocampal pyramidal cells). At later time points (P20 and P10m), numerous interneurons and principal neurons expressed TLR8 (e, g). Labeling intensity of PV-interneurons at these stages was stronger than in very young mice and the numbers of PV-immunoreactive interneurons was increased (f, h). Abbreviations: DG, dentate gyrus; CA1, CA2, CA3, sectors CA1-CA3 of the hippocampus proper. The dashed lines in h demarcate the anatomical borders of sector CA2.

Animal experiments were conducted according to national guidelines and European Community laws and were approved by the Committee for Animal Protection of the Austrian Federal Ministry of Education, Science and Research. C57BL/6J wild-type mice were obtained from Charles River (Sulzfeld, Germany). They were housed in groups of up to five mice in single-ventilated cages with a twelve hours light/dark cycle (lights were turned on at 6:30 am) and had access to food and water *ad libitum*. For immunohistochemistry experiments, 18 mice from postnatal days 5 (n = 2), 10 (n = 2) and 20 (n = 2), 10 to 14 weeks old young adult mice (n = 8), and 10 months (n = 4) old male and female mice were used (see Figs 1–7 and S1 Fig). For qPCR, 10 days old mice (n = 4) were used (S1 Protocol and S2 Fig).

### Perfusion and cutting

Adult mice were injected intraperitoneally with an overdose of thiopental (150 mg/kg) and perfused transcardially with phosphate-buffered saline (PBS) at room temperature for three minutes (pump speed: 9 ml/min) followed by perfusion with ice-cold 4% paraformaldehyde (PFA) in 50 mM PBS (pH 7.4) for 10 minutes. Mouse pups were perfused transcardially using a 50 mL syringe. The brains were removed from the skulls and post-fixed for another 90 minutes in 4% PFA (4°C) followed by immersion in 20% sucrose in 50 mM PBS at 4°C for 24 hours. Finally, brains were snap-frozen in isopentane (-70°C, 3 minutes; Merck, Darmstadt, Germany), kept in open vials at -70°C for 24 hours, and finally stored in tightly sealed vials at -20°C for up to two weeks. Finally, coronary sections (40 μm) were cut on a cryostat-microtome, collected in 50 μM Tris-buffered saline (TBS, pH 7.2) containing 0.1% sodium azide (Merck, Darmstadt, Germany), and stored at 4°C.

### Fluorescence immunohistochemistry

The immunohistochemical studies were performed on free-floating sections after being matched anatomically across all animals. Six double labeling experiments for TLR8 (toll-like receptor 8) and the following glial and neuronal markers were performed: GFAP (glial fibrillary acidic protein), TMEM-119 (transmembrane protein 119), NeuN (Fox-3, neuronal nuclear protein), PV (parvalbumin), SOM (somatostatin), and CR (calretinin). The used antibodies for each reaction are listed in Table 1. The sections were first incubated with Tris-HCl-buffered saline (TBS; Margaritella, #BT1007), containing 0.4% TritonX-100 (TBS-Triton; Merck, #1.08603) for 30 min and then with the blocking serum (10% normal horse serum in TBS-Triton) for 90 minutes. For the experiments with antibodies raised in mouse (GFAP, NeuN, PV, and SOM) sections were incubated with a “Mouse on Mouse” blocking reagent (Vector, #MKB-2213) diluted 1:27.7 in TBS for 60 min. After washing the sections with TBS-Triton, they were incubated overnight at room temperature with the primary antibodies (against TLR8 and the second marker), which were diluted in the blocking serum (containing 0.1% sodium azide) according to Table 1. After washing three times for five minutes with TBS-Triton, the sections were incubated with the secondary antibodies for 120 min (see Table 1). TLR8 was labeled using tyramide signal amplification (TSA) and 5-(and-6)-carboxytetramethylrhodamine (TAMRA, 1:2000), except for the double labeling with GFAP, where TSA and Fluorescein (1:300; Thermo Scientific, #46410) was used. PV, SOM, and NeuN were visualized using streptavidin 649 (1:100 in TBS). CR was visualized with TSA and CY5 (1:500). GFAP did not need a secondary antibody and staining, since we used a CY3-conjugated primary antibody. All staining solutions using TSA contained 0.5% hydrogen peroxide (1:166; Merck, #1.07209), 4-Iodophenylboronic acid (4-IPBA, 1:1000), TWEEN 20 (1:1000) and 50% dextran sulfate (1:25). The double labeling experiment with CR differed in its sequence from the rest, because the two labeling procedures were not carried out simultaneously, but successively with an additional blocking step in between. For CR, 20 minutes blocking using 100 mM sodium sulfate was performed. Afterwards, the sections were mounted on glass slides using gelatin and covered with Vectashield mounting medium (Szabo Scandic/Vector, #H-1000).

**Table 1:** Origin and concentrations of primary and secondary antibodies used in the study.

**Primary AB**	**Species**	**Manufacturer**	**Catalogue number**	**Dilution**
TLR8	Rabbit	Sigma	A31768	1:1000
GFAP	Mouse	Sigma	C9205	1:1000
TMEM-119	Rabbit	Synaptic Systems	#400002	1:500
NeuN	Mouse	Millipore	MAB 377	1:5000
Parvalbumin	Mouse	Sigma	P3088	1:5000
Somatostatin	Mouse	Santa Cruz	Sc 55565	1:1000
Calretinin	Goat	Millipore	AB1550	1:10000
**Secondary AB**				
Anti-Rabbit	Donkey	Invitrogen	A16023	1:500
Anti-Mouse biotin.	Horse	Vectorlabs	BA-2000	1:200
Anti-Goat	Donkey	Invitrogen	A15999	1:500

### Quantification of colocalization

To quantify hippocampal interneurons co-expressing TLR8 together with markers for GABAergic interneurons (PV, SOM, CR), microphotographs of immunolabeled sections obtained from male 10 to 14 weeks old mice were taken at 20-fold primary magnification using a fluorescence microscope (Imager.M1, Carl Zeiss, Germany). Images were imported into NIH ImageJ 1.53c (National Institutes of Health, Bethesda, MD, USA), and photographs of the individual channels were displayed side by side. Interneurons were discriminated from glutamatergic principal cells based on their size and shape that clearly differed from the uniform size and shape of principal neurons. Using the “Cell Counter” plugin, the numbers of single-labeled and double-labeled cells were determined. In total, 3,493 labeled neurons (TLR8, 1,645; PV, 1,249 (N = 8); SOM, 449 (N = 3); CR, 141 (N = 2)) from up to 8 mice were analyzed to identify co-labeling with other markers.

### Statistical analysis

Statistics were calculated using GraphPad Prism v.8.4.2 (GraphPad Software, San Diego, USA) and values are given as mean ± SEM.

## Results

### Expression of TLR8 in the dentate gyrus

#### Distribution of TLR8-immunoreactive cell bodies in the dentate gyrus

As shown in Fig 1A–1D and 1G, TLR8-immunoreactive cells in the dentate gyrus showed a highly variable labeling intensity. While dentate granule cells showed only weak TLR8-immunoreactivity, other cells in and around the granule cell layer (presumed GABAergic interneurons) were intensely labeled. In the hilus of the dentate gyrus, many cells with intermediate labeling intensity were present (presumed GABA interneurons and hilar mossy cells). Only very few TLR8-positive neuronal cell bodies were observed in the middle and outer molecular layer of the dentate gyrus. Double immunofluorescence of TLR8 and the microglia marker transmembrane protein 119 (Tmem-119; Fig 1A–1C) did not reveal double-labeled cells. Also, double-labeling of TLR8 with the astrocyte marker glial fibrillary acidic protein (GFAP; Fig 1A–1C) did not reveal double-positive cells. In contrast, double-labeling of TLR8 and the neuronal marker neuron-specific nuclear protein (NeuN; Fig 1G–1I) provided evidence for expression of TLR8 in many neurons.

#### Expression of TLR8 in GABAergic interneurons of the dentate gyrus

To investigate if TLR8 is expressed in specific subgroups of GABAergic interneurons of the dentate gyrus, double-immunofluorescence of TLR8 with the calcium-binding proteins parvalbumin (PV) and calretinin (CR) and with the neuropeptide somatostatin (SOM) were performed. As shown in Fig 2A–2C, many PV-immunoreactive interneurons were also labeled by the TLR8 antibody. Most of them were neurons with a relatively large cell body located in the hilus of the dentate gyrus and in and around the granule cell layer (presumed basket- and axo-axonic cells). Co-labeling of TLR8 with SOM revealed a lower fraction of double-labeled neurons (Fig 2D–2F). The distribution of SOM-immunoreactive interneurons was restricted to the hilus of the dentate gyrus (Fig 2E). Co-labeling of TLR8 with CR, a marker for interneuron-specific interneurons, revealed almost no colocalization (Fig 2G–2I). Quantification of colocalization revealed that 63.4 ± 5.70% of TLR8-expressing interneurons also expressed PV, but only 6.7 ± 6.67% and 0.9 ± 0.86% expressed SOM and CR, respectively (Fig 2J). On the other hand, 87.7 ± 4.04% of PV-interneurons co-expressed TLR8, but only 2.9 ± 2.86% of SOM-interneurons and 3.1 ± 3.13% of CR-interneurons co-expressed TLR8 (Fig 2K).

### Expression of TLR8 in hippocampal sector CA3

#### Distribution of TLR8-immunoreactive cell bodies in sector CA3

In sector CA3 of the hippocampus proper, TLR8-immunoreactive cells consisted of weakly labeled pyramidal cells in the pyramidal cell layer and of more intensely labeled neurons in stratum oriens, pyramidale, lucidum, radiatum, and at the border between stratum radiatum and stratum lacunosum-moleculare (Fig 3A–3D and 3G). While double immunohistochemistry of TLR8 with the glial markers Tmem-119 (microglia; Fig 3A–3C) and GFAP (astrocytes; Fig 3D–3F) showed no cellular colocalization, immunohistochemistry together with NeuN (neurons; Fig 3G–3I) revealed that virtually all TLR8-positive cells in sector CA3 were neurons.

#### Expression of TLR8 in CA3 GABA-interneurons

Similar as in the dentate gyrus, the majority of intensely TLR8-labeled interneurons also expressed PV (Fig 4A–4C). PV-immunoreactive fibers (presumed axons of PV-expressing basket- and axo-axonic cells) surrounded the cell bodies of TLR8-positive principal neurons in the pyramidal cell layer of sector CA3 (Fig 4B and 4C). Double-immunohistochemistry of TLR8 together with SOM (Fig 4D–4F) revealed co-expression of TLR8 with SOM-positive interneurons in stratum oriens, but not in stratum pyramidale, radiatum, lucidum, and lacunosum-moleculare. As shown in Fig 4G–4I, TLR8 was only rarely co-expressed in CR-positive interneurons. Quantification of colocalization revealed that 67.0 ± 7.49% of TLR8-interneurons also expressed PV, 28.9 ± 4.19% co-expressed SOM, but only 1.9 ± 1.92% co-expressed CR (see Fig 4J). On the other hand, 64.0 ± 4.56% of PV-interneurons, 10.7 ± 1.72% of SOM-interneurons, and only 2.9 ± 2.94% of CR-interneurons co-expressed TLR8 (see Fig 4K).

### Expression of TLR8 in hippocampal sector CA1

#### Distribution of TLR8-immunoreactive cell bodies in sector CA1

In hippocampal sector CA1, weak and uniform TLR8-immunolabeling was observed in cell bodies of pyramidal cells in stratum pyramidale (see Fig 5A–5D and 5G). More intensely labeled somata of presumed interneurons were present in stratum oriens, pyramidale, radiatum, and lacunosum-moleculare. Double-labeling with Tmem-119 and GFAP showed a lack of expression of TLR8 in microglia (Fig 5A–5C) and astrocytes (Fig 5D–5F), respectively. In contrast, almost all TLR8-immunoreactive cells were also positive for the neuronal marker NeuN (see Fig 5G–5I).

#### Expression of TLR8 in CA1 GABA-interneurons

In hippocampal sector CA1, TLR8 was expressed in the majority of PV-expressing interneurons (see Fig 6A–6C). Most PV-immunoreactive interneurons were located in and around the pyramidal cell layer and many PV-positive axons innervated cell bodies of pyramidal cells in stratum pyramidale. In contrast to the expression pattern observed in hippocampal sector CA3, TLR8 was only rarely co-expressed in SOM-positive interneurons in sector CA1 (Fig 6D–6F). SOM-labeled neuronal cell bodies were mainly located in stratum oriens (Fig 5E). Colocalization of TLR8 and CR was only rarely detected in sector CA1 (Fig 6G–6I). Quantification of double-labeled cells showed that 79.9 ± 5.47% of TLR8-interneurons also expressed PV, but only 1.1 ± 1.11% co-expressed SOM, and 1.3 ± 1.32% co-expressed CR (see Fig 6J). Moreover, quantification of co-labeling revealed that 56.8 ± 8.67% of PV-interneurons, 0.6 ± 0.61% of SOM-interneurons, and 1.4 ± 1.39% of CR-interneurons of sector CA1 co-expressed TLR8 (Fig 6K).

### Postnatal development of TLR8-expression

To investigate the postnatal time course of expression of TLR8 and PV in the hippocampus, we immunolabeled brains from P0, P5, P10, and P20 pups and from adult mice (10 months, P10m). At P0, neither TLR8- nor PV-immunoreactive cells were detected in the hippocampus and dentate gyrus (not shown). As shown in Fig 7A and 7B, weak expression of TLR8, but not of PV, was already present in small numbers of neurons in the hilus of the dentate gyrus and in the hippocampus proper at P5. Five days later (at P10, Fig 7C), expression of TLR8 was detected in the principal cell layers (granule cell layer of the dentate gyrus and pyramidal cell layer of the hippocampus proper) and in presumed GABAergic interneurons located in the inner molecular layer, granule cell layer, and hilus of the dentate gyrus, in strata oriens, pyramidale, lucidum (CA3), radiatum, and lacunosum-moleculare of the hippocampus. At this developmental stage, we also detected PV-immunoreactive interneurons in strata oriens, pyramidale and radiatum of the hippocampus and in the hilus of the dentate gyrus (Fig 7D). Expression of TLR8 at P20 (Fig 7E) already closely resembled the expression in the adult mouse (P10m, Fig 7G) and was characterized by faint TLR8-labeling in dentate granule cells and pyramidal cells in hippocampal sector CA1 and a stronger labeling in the pyramidal cell layer of sector CA3. Numerous neurons (presumably GABAergic interneurons and glutamatergic mossy cells) were positive for TLR8 in the hilus of the dentate gyrus (see Fig 7E and 7G). Several neurons with a more intense labeling were present in the granule cell layer of the dentate gyrus and also in the pyramidal cell layers of hippocampal sectors CA3 and CA1. In sector CA3, many intensely labeled interneurons with large somata were detected in stratum oriens, pyramidale, and lucidum. The major difference between P20 and P10m was the presence of several TLR8-immunoreactive interneurons in stratum radiatum and at the border between stratum radiatum and stratum lacunosum-moleculare at P20 (compare Fig 7E and 7G). At P10m, TLR8-positive interneurons were mainly concentrated in stratum oriens and pyramidale of sector CA1. Expression of PV was more intense at P20 and P10m than at P5 and higher numbers of interneurons expressing PV were present at these developmental stages. At P10m, the perisomatic innervation of principal neurons by PV-immunoreactive axons was more intense than at earlier time points (see Fig 7F and 7H).

## Discussion

In this study, we applied double-staining immunohistochemistry to assess the co-expression of TLR8 in diverse neuronal subtypes throughout the different developmental stages of the mouse hippocampus, observing a remarkable congruent expression of TLR8 with PV from the postnatal day 10 to the adult 10 months old mice. A lower degree of colocalization was detected in SOM-expressing interneurons. Only very sparse colocalization of CR and TLR8 was detected. In addition, a weak expression of TLR8 was found in the granule cell layer of the dentate gyrus and pyramidal cell layer of the hippocampus proper in all developmental stages after P5. Together with the highly cell type-specific expression of TLR8 in the mouse hippocampus, these data suggest an important and distinct role of TLR8 in the development of PV- (and SOM-) expressing interneurons.

GABAergic inhibitory interneurons are specialized cells with diverse subtypes, regulating complex network functions by targeting pyramidal neurons and other interneurons. In this study, we focused on the three big subtypes of interneurons defined by the expression of either PV, SOM or CR, estimated to comprise almost 23, 11, and 14% of the CA1 interneurons, respectively [19–21]. PV-expressing interneurons include mainly basket cells, axo-axonic cells, as well as bistratified cells, originating from the medial ganglionic eminence (MGE) [22]. While the former two groups innervate the soma and axon-initial segment of pyramidal cells (PCs), respectively, and almost exclusively express PV, bistratified cells target PC dendrites and co-express SOM [19,23]. It has been shown that in rodents, PV expression is very low before P10 and then increases to mature levels between P12-P30 [24], which is in accordance to our result that showed a lack of PV positive cells in P0 and P5 pups. A fraction of oriens-lacunosum moleculare (O-LM) cells also seem to express PV, although at significantly lower levels [25,26]; however, the defining molecular marker of them is SOM [19]. O-LM cells appear to have dual MGE and CGE (caudal ganglionic eminence) origin [27] and have a well-defined anatomy, with soma and dendrites restricted to stratum oriens, while their axon densely collateralizes within stratum lacunosum-moleculare where it innervates distal dendrites of pyramidal cells [28]. In contrast to PV or SOM expressing interneurons, CR-expressing interneurons preferentially innervate other GABAergic interneurons, providing a cellular substrate specialized for network disinhibition [19]. This group contains interneuron selective interneurons (ISIs) type one and three [20], and originates in the CGE [22]. Based on the location and marker profile of the TLR8 expressing cells in our study, the interneurons co-expressing TLR8 together with SOM and PV are probably bistratified and O-LM cells. They are both SOM- and PV-positive dendritic inhibitory interneurons located within stratum oriens. Moreover, we detected coexpression of TLR8 and PV in interneurons in layers that are devoid of SOM-immunoreactive interneurons, most likely representing PV-expressing basket cells and axo-axonic cells.

Previous reports indicate that inflammatory processes and neuroinflammation play a key role in the clinical outcome of epilepsy [29,30] and TLR pathways might mediate this connection as prototypical inflammatory pathways [31,32]. Hippocampal PV-expressing interneurons seem to be one of the most vulnerable neuronal groups in human temporal lobe epilepsy [33] as well as in animal models of epilepsy [34,35] and their dysfunction or degeneration may contribute to epileptogenesis [36]. Given the high expression levels of TLR8 in PV-interneurons and the fact that robust activation of TLR8 in neurons can induce neuronal death [17], it is tempting to speculate that non-canonical TLR8-signaling in hippocampal PV-interneurons may contribute to their degeneration in human epilepsy and in animal epilepsy models based on an initial status epilepticus or traumatic brain injury.

Lucchi et al. showed that EP-80317, a ghrelin receptor antagonist, was able to activate PPARγ, which led to anticonvulsant activity [37]. This hypothesis has been backed by other studies, which demonstrated that treatment with PPARγ agonists (e.g. rosiglitazone, pioglitazone) induces an anticonvulsant effect [38,39]. Further, there seems to be a correlation between the expression levels of PPARγ and TLR8. In the previously mentioned study by Lucchi et al, the authors observed elevated expression of PPARγ in the same group of interneurons herein described for TLR8 (i.e. SOM, PARV) and Balada et al. [40] showed that in patients with neonatal encephalopathy the expression of PPARγ and TLR8 (during the first 4 days of life) was increased in relation to controls. Considering the above data, it would be interesting to test the hypothesis whether treatment of epileptic mice with PPARγ ligands (e.g. rosiglitazone) would exert anti-epileptic effects due to downregulation of TLR8 expression in SOM and PV neurons, or whether this intervention would functionally modulate the inflammatory activity mediated by TLR8 ligation. Such a PPARγ-regulating mechanism, ligand-dependent transrepression of transcription factors, has been described in the case of immune cells [41], and would open the door to the repurposing of type II diabetes mellitus drugs for their use in epilepsy.

TLR8 has been shown to use an independent signaling pathway in neurons, making it likely for TLR8 to have another function added to the immunological one. Since TLR8 expression is diminished in an adult brain compared to embryogenesis, Ma et al. (2007) proposed switching the role of TLR8 function from developmental to immunological as mice grow up [17]. In the mature brain, TLR8 might be quiescent under normal conditions, but is being activated in response to adequate stimuli, like tissue injuries or viral infection, and may induce according responses in neuronal morphology. In accordance with the non-immunological function of TLR8 in neurons, by combining *in vitro* neuronal cultures, *in utero* electroporation, and transcriptomics, Hung et al. (2018) found the promotion of dendritic pruning and dendritic shortening upon the activation of TLR8. In addition, its activation also increases dendritic spine density. They reported that TLR3, TLR7 and TLR8 regulate neuronal morphogenesis through complex and varying pathways [42]. Such a mechanism could also contribute to the plasticity of the hippocampal network in adult mice. To the best of our knowledge, there exists no study assessing the presence and the role of TLR8 in the adult rodent hippocampus, restraining us from a comparison of our data with it. To fully understand the possible role of TLR8 in the hippocampus of adult mice, implementing TLR8 knockout mice could be helpful.

In conclusion, while many questions regarding neuronal TLR8 functionality and intracellular signaling remain unanswered, our data provide evidence of a specific expression of TLR8 in PV- and SOM-expressing hippocampal interneurons, which might suggest a role for TLR8 in the adult mouse hippocampus, beyond the immune responses.

## Supporting information

S1 Fig Assessment of TLR8 expression in PV expressing interneurons of different brain regions. Double immunofluorescence of TLR8 (red fluorescence; a, d, g, j, m) with parvalbumin (PV; b, e, h, k, n). TLR8 was predominantly expressed in PV-positive interneurons in cortex and globus pallidus, but no colocalization was found in medial septum, caudate putamen and thalamus.
(TIF)

S2 Fig Gene expression of TLR8 in hippocampus and cortex of ten days old pups. In hippocampus, in comparison to the whole tissue (containing glial cells and neurons), TLR8 was mainly expressed in neurons. In cortex, neurons have only a tiny portion of the expression.
(TIF)

S1 Protocol Supporting methods: RNA extraction and qPCR. (DOCX)
