# Supplementary figures and images for: Expression of toll like receptor 8 (TLR8) in specific groups of mouse hippocampal interneurons
Source: PLoS One. 2022 May 4;17(5):e0267860. doi: 10.1371/journal.pone.0267860 (PMC9067651; doi:10.1371/journal.pone.0267860)

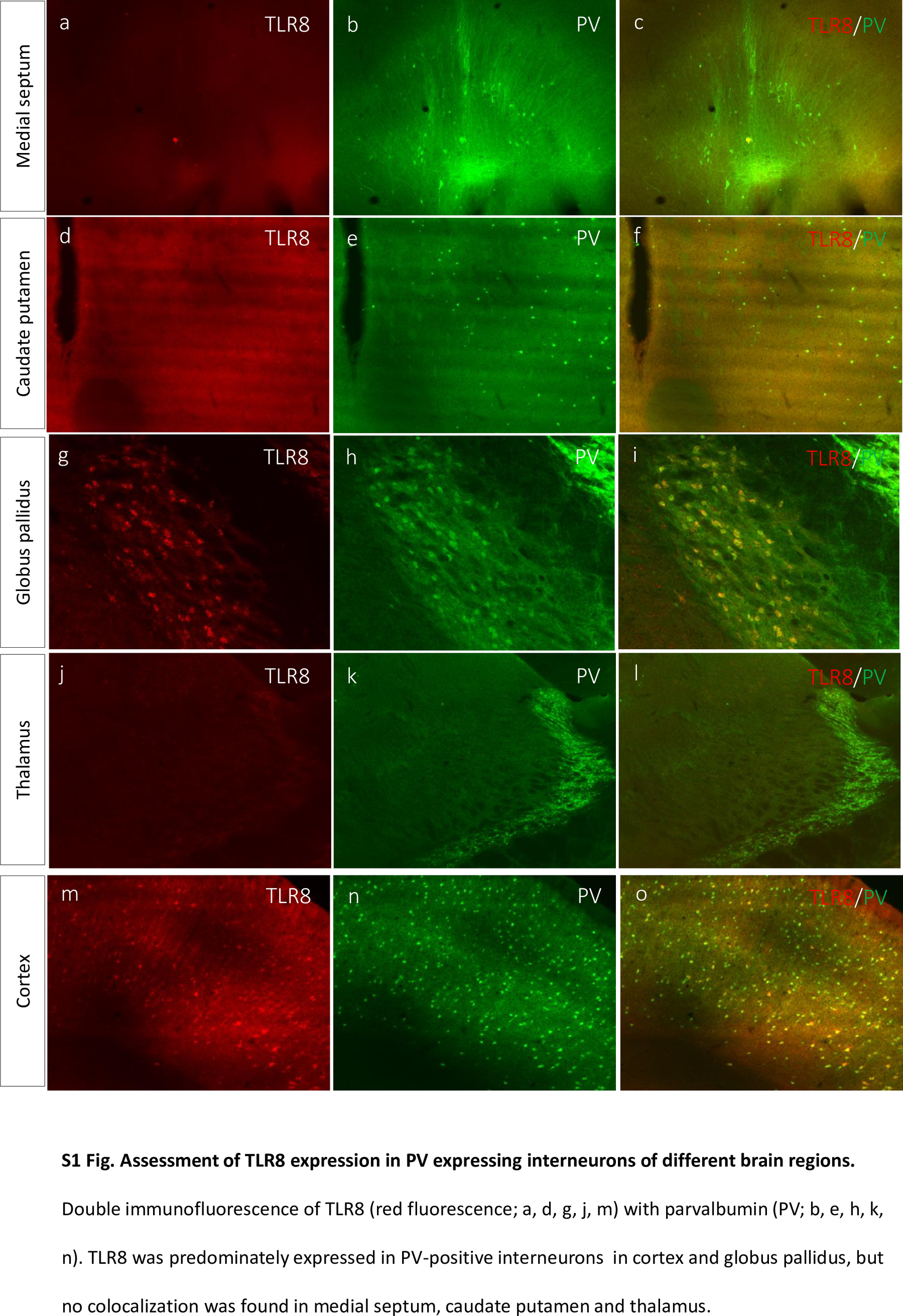

Supplement: S1 Fig — Double immunofluorescence of TLR8 (red fluorescence; a, d, g, j, m) with parvalbumin (PV; b, e, h, k, n). TLR8 was predominantly expressed in PV-positive interneurons in cortex and globus pallidus, but no colocalization was found in medial septum, caudate putamen and thalamus. (TIF) [file pone.0267860.s001.tif]

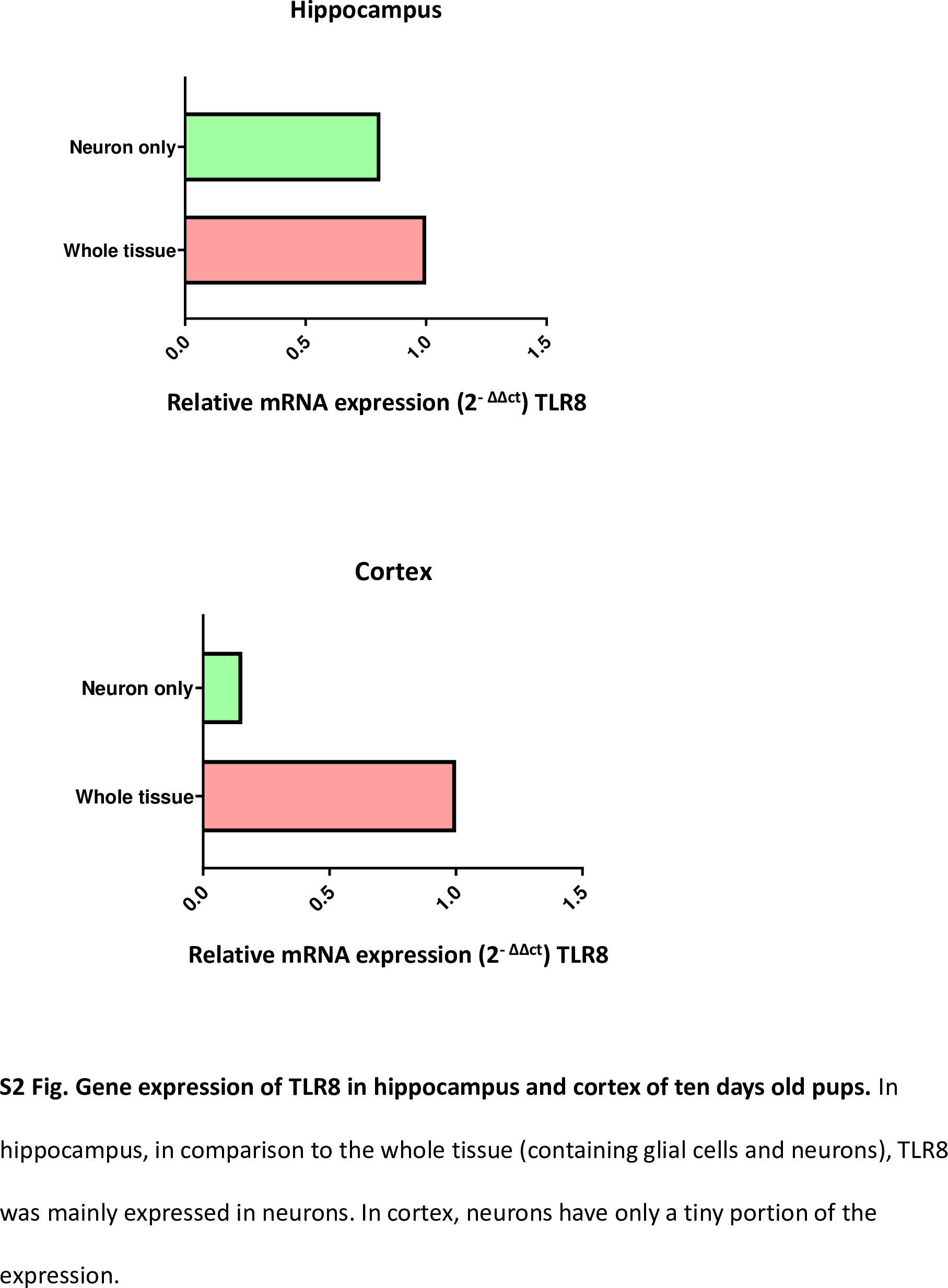

Supplement: S2 Fig — In hippocampus, in comparison to the whole tissue (containing glial cells and neurons), TLR8 was mainly expressed in neurons. In cortex, neurons have only a tiny portion of the expression. (TIF) [file pone.0267860.s002.tif]
