# Supplementary material for: Expression of toll like receptor 8 (TLR8) in specific groups of mouse hippocampal interneurons
Source: PLoS One. 2022 May 4;17(5):e0267860. doi: 10.1371/journal.pone.0267860 (PMC9067651; doi:10.1371/journal.pone.0267860)
Supplement: S1 Protocol — (DOCX) [file pone.0267860.s003.docx]

**Supporting Methods**

**RNA extraction**

In order to extract RNA from mouse (n = 2) brain tissue, cortex and hippocampi were dissected and placed in 300 µl of DNA/RNA Protection Reagent containing Proteinase K (Cell Signaling Technology). The samples were mixed with sterile glass beads and they were homogenized by vortexing. Afterwards, the RNA preparation was done with the kit Monach Total RNA Miniprep Kit (Cell Signaling Technology) according to the manufacturer´s instructions.

To isolate RNA specifically from neuronal cells, neurons from 10 days old mice (n = 2) were prepared according to Brewer et al. [1]. Shortly, hippocampi and cortex were separated from the rest of the brain regions, they were placed in NeurobasalA media plus B27 supplement (Thermo Fischer Scientific) and they were immediately trypsinized (Sigma Aldrich) at 37°C for 15 min. Afterwards, neurons were separated from microglia and oligodendrocytes by means of a density gradient (Optiprep, Sigma Aldrich). The isolated neurons were lysed and RNA was extracted with the Monarch Total RNA Miniprep Kit.

**qPCR**

Reverse transcription of 450 ng of RNA was performed with the LunaScript RT Supermix kit (Cell Signaling Technology) in a 20 µl reaction volume. Expression of TLR8 (Mm04209873_m1) and the housekeeping gene Rpl13a (Mm01612986-gH) was done with the indicated TaqMan primers (ThermoFischer Scientific) and the Luna Universal Probe qPCR Master Mix (Cell Signaling Technology). Since the expression of TLR8 is observed in whole tissue (contains glia cells and neurons) of 10 days old pups [2], we normalized the expression of TLR8 in neurons in comparison to the whole tissue using the δδCT approximation method.

**References**

1. Brewer GJ. Isolation and culture of adult rat hippocampal neurons. J Neurosci Methods 1997; 71(2):143–55.

2. Kaul D, Habbel P, Derkow K, Krüger C, Franzoni E, Wulczyn FG et al. Expression of Toll-like receptors in the developing brain. PLoS ONE 2012; 7(5):e37767.
